# Insidious Synovial Sarcoma of Bone in a Patient with Rheumatoid Arthritis

**DOI:** 10.1055/s-0041-1739172

**Published:** 2021-11-11

**Authors:** Juan Carlos Reyes Villarreal, Luis Francisco Pineda-Galindo, Olga Vera-Lastra, Elizabeth Natalia Quispe-Susara, Alberto Ordinola Navarro

**Affiliations:** 1 Departamento de Medicina Interna, Hospital de Especialidades "Dr. Antonio Fraga Mouret,"Centro Médico Nacional La Raza del Instituto Mexicano del Seguro Social, Cidade do México, México; 2 Departamento de Patologia, Hospital de Especialidades "Dr. Antonio Fraga Mouret,"Centro Médico Nacional La Raza del Instituto Mexicano del Seguro Social, Cidade do México, México

**Keywords:** arthritis, rheumatoid, sarcoma, synovial, soft tissue neoplasms

## Abstract

Synovial sarcoma is a rare malignity of mesenchymal origin; the diagnostic approach usually begins by documenting a soft tissue tumor; however, it results in a challenging diagnosis when it is more profound, of small size, or primary from the bone.

The present report describes a patient who presented insidious onset hip pain attributed to rheumatoid arthritis, with a fatal outcome due to baseline disease and surgery complications.

The underestimation of hip pain, mainly when there is no palpable mass, may delay the diagnosis.

## Introduction

Synovial sarcomas (SSs) are a rare mesenchymal neoplasm with capacities of dual differentiation; a periarticular soft-tissue mass is the most common presentation; however, it can arise from any site. 1 2 The underestimation of hip pain, mainly when there is no palpable mass or a soft-tissue tumor in imaging studies, may delay the diagnosis.

## Case Report

A 67-year-old man with a 20-year history of rheumatoid arthritis (RA) started presenting with hip pain; he was evaluated by different departments, and the symptoms were attributed to RA and just symptomatic treatment was indicated.

**Fig. 1:**
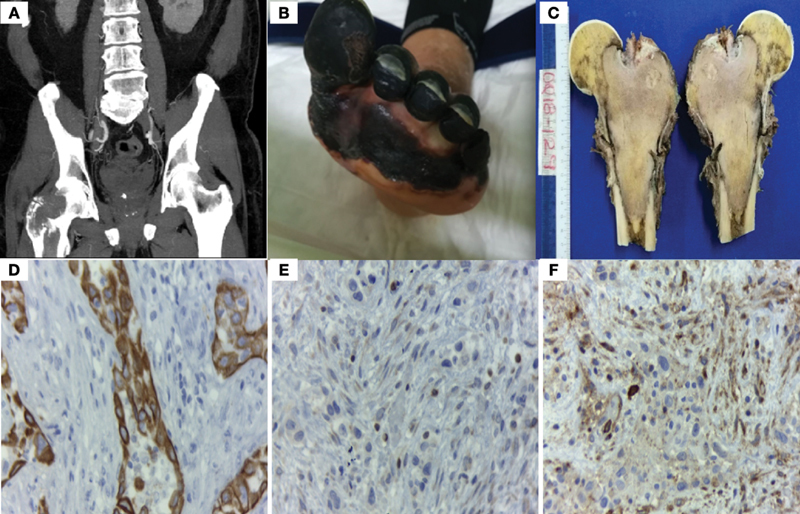
(A) Computed tomography scan showing osteolytic lesion in the neck and head of the femur. (B) Deep venous thrombosis with distal necrosis. (C) Right femoral neck and head showing intraosseous infiltration. (D-F) Immunohistochemistry. The identification of CK7 (D), BCL-2 (E), and CD99 (F) were the key in suspecting the diagnosis of synovial sarcoma.

A year later, he was referred to our hospital due to persistence of pain and edema in the lower limbs. A computed tomography (CT) scan was performed on hospitalization, reporting an osteolytic lesion in the neck and head of the femur ( Fig. 1A ). He developed deep venous thrombosis, which evolved into necrosis of the left toe; significant laboratory tests included positive serum cryoglobulins (polyclonal IgG and monoclonal IgM), elevated rheumatoid factor and hypocomplementaemia ( Fig. 1B ). He went to the operating room, where the right femoral head was resected and a proximal femur megaprosthesis was placed ( Fig. 1C ). During the postoperative period, the patient developed an infection at the surgical site; he was treated with antibiotics, wound cleansing and debridement. The histological diagnosis revealed: epithelial and spindle cells positive for cytokeratin 7, BCL2 and CD99. Molecular studies with translocation t(X;18) (p11.2; q11.2), and SS18-SSX1 fusion proteins expression, corroborated biphasic the diagnosis of SS. However, the patient had complications with cerebrovascular disease and pulmonary thromboembolism and died.

## Discussion

The present report describes a patient who presented insidious onset hip pain attributed to RA, with a fatal outcome due to baseline disease and surgery complications.

Synovial sarcoma is a rare malignity of mesenchymal origin that comprises approximately up to 10% of soft tissue sarcomas. It may present in any anatomic site, but SS arising from soft tissue is the most common presentation. 1 Synovial sarcoma as a primary bone tumor is relatively rare, and it is more frequent in young adult patients. 2

Synovial sarcoma was initially described as a biphasic neoplasm comprising both epithelial and spindle cell components; however, histologic variants such as monophasic, biphasic, and poorly differentiated have been identified. 1 2 According to the reported series, the main symptoms are neuropathic and local pain, motor deficit, and palpable mass; in our patient, the insidious symptomatology and history of rheumatic disease delayed the diagnosis. 3

The diagnostic approach usually begins by documenting a soft tissue tumor; when it is superficial, it is easy to identify it; however, when it is more profound, of small size, or primary from bone, imaging studies showing internal hemorrhage, calcification or osteolytic lesions can help us to guide our diagnostic suspicion. 2 In our patient, a CT scan was performed, in which no tumor was found; however, a right femoral head osteolytic lesion was reported, which led to the suspicion of bone tumor.

Immunohistochemistry is essential in suspecting the diagnosis. In this patient, CK7, CD99 and BCL2 enabled suspecting the diagnosis and classifying the tumor as biphasic, since CK7 is found in epithelial cells and CD99, along with BCL2, are found in spindle cells ( Fig. 1 D-F ). 4

Synovial sarcoma has a pathognomonic translocation between chromosomes X and 18, t (X;18) (p11.2;q11.2), translating into several different expressions of SS18:SSx fusion proteins; with SS18-SSX1 and SS18:SSX2 being the most common. After immunohistochemistry, it was possible to corroborate the diagnosis demonstrating the translocation and fusion proteins expression; the fusion is detectable in > 95% of cases, being a key tool to confirm the diagnosis. 1 4

Metastatic disease is present in 24% of patients at the time of diagnosis; the prognosis for patients without metastasis is favorable. However, metastasis occurs in 50 to 70% of cases, and most develop in the lungs, followed by bone and liver. The standard treatment is surgical resection for localized SSs with consideration of using adjuvant radiation and/or systemic anticancer therapy. However, there is no standard approach for the use of systemic therapy. When prescribed, anthracycline plus ifosfamide are usually the first-line therapy. Nevertheless, future clinical trials would define an appropriate treatment for this disease. 5 The knowledge of the disease can help to guide immunohistochemistry, corroborating the diagnosis and starting an individualized therapy that leads to better outcomes.
