# Supplementary material for: Insidious Synovial Sarcoma of Bone in a Patient with Rheumatoid Arthritis
Source: Rev Bras Ortop (Sao Paulo). 2021 Nov 11;59(Suppl 1):e31–3. doi: 10.1055/s-0041-1739172 (PMC11254417; doi:10.1055/s-0041-1739172)
Supplement: Related texts

# Research article: Sarcoma sinovial insidioso de osso em um paciente com artrite reumatoide

**Keywords:** artrite reumatoide, neoplasias de tecidos moles, sarcoma sinovial

## Abstract

Sarcoma sinovial é uma rara malignidade de origem mesenquimal; a abordagem diagnóstica geralmente começa documentando um tumor de tecido mole; no entanto, resulta em um diagnóstico desafiador quando é mais profundo, de pequeno porte ou primário do osso.

O presente relato descreve um paciente que apresentou dor insidiosa no quadril atribuída a artrite reumatoide, com desfecho fatal devido a doenças de base e complicações cirúrgicas.

A subestimação da dor no quadril, principalmente quando não há massa palpável, pode atrasar o diagnóstico.

## Introdução

Os sarcomas sinoviais (SSs) são uma rara neoplasia mesenquimal com capacidades de dupla diferenciação; uma massa de tecido mole periarticular é a apresentação mais comum; no entanto, eles podem surgir de qualquer local. 1 2 A subestimação da dor no quadril, principalmente quando não há massa palpável ou um tumor de tecido mole em estudos de imagem, pode atrasar o diagnóstico.

## Relato de Caso

Um homem de 67 anos com 20 anos de história de artrite reumatoide (AR) começou com dor no quadril; ele foi avaliado por diferentes departamentos, sendo os sintomas atribuídos a AR e indicando apenas tratamento sintomático.

Um ano depois, ele foi encaminhado ao nosso hospital devido à persistência de dor e edema dos membros inferiores. Foi realizada tomografia na internação, relatando lesão osteolítica no colo e na cabeça do fêmur ( Fig. 1A ). O paciente desenvolveu trombose venosa profunda, a qual evoluiu para necrose do dedão do pé esquerdo; testes laboratoriais significativos incluíram crioglobulinas soropositivas (monoclonal e igM monoclonal) elevados, fator reumatoide elevado e hipocomplementaemia ( Fig. 1B ). Ele foi para a sala de cirurgia, onde a cabeça do fêmur direito foi resseccionada, sendo colocada uma mega próstese proximal do fêmur ( Fig. 1C ). Durante o pós-operatório, o paciente desenvolveu uma infecção no sítio cirúrgico; ele foi tratado com antibióticos, limpeza de ferida e debridamento. Diagnóstico histológico revelado: células epiteliais e fusos positivas para citoqueratina 7, BCL2 e CD99. Estudos moleculares com expressão de proteínas de fusão translocação (X;18) (p11.2; q11.2) e expressão de proteínas de fusão SS18-SSX1 corroboraram o diagnóstico de SS bifásico. No entanto, ele se complicou com doença cerebrovascular e tromboembolismo pulmonar e morreu.

**Fig. 1:** (A) Tomografia computadorizada mostrando lesão osteolítica no colo e na cabeça do fêmur. (B) Trombose venosa profunda com necrose distal. (C) Colo e cabeça femorais direitas mostrando infiltração intraóssea. (D-F) Imunohistoquímica. A identificação de CK7 (D), BCL-2 (E) e CD99 (F) foi a chave para suspeitar do diagnóstico de sarcoma sinovial.

## Discussão

O presente relatório descreve um paciente que apresentou dor insidiosa no quadril atribuída a AR, com desfecho fatal devido a doenças de base e complicações cirúrgicas.

O SS é uma malignidade rara de origem mesenquimal que compreende ∼ 10% dos sarcomas de tecido mole. Pode apresentar-se em qualquer local anatômico, mas decorrente de tecido mole é a apresentação mais comum. 1 O SS como um tumor ósseo primário é relativamente raro e é mais frequente em pacientes adultos jovens. 2

O SS foi inicialmente descrito como uma neoplasia bifásica composta por componentes epiteliais e células de fuso; no entanto, foram identificadas variantes histológicas como monofásica, bifásica e pouco diferenciada. 1 2 De acordo com a série relatada, os principais sintomas são dor neuropática e local, déficit motor e massa palpável; em nosso paciente, a sintomatologia insidiosa e a história da doença reumática atrasaram o diagnóstico. 3

A abordagem diagnóstica geralmente começa documentando um tumor de tecido mole; quando é superficial, é fácil identificá-lo; no entanto, quando é mais profundo, de pequeno porte ou primário do osso, estudos de imagem mostrando hemorragia interna, calcificação ou lesões osteolíticas podem nos ajudar a guiar nossa suspeita diagnóstica. 2 No nosso paciente, foi realizada a tomografia computadorizada, na qual não foi encontrado tumor; no entanto, foi relatada uma lesão osteolítica da cabeça femoral direita, o que levou à suspeita de tumor ósseo.

A imunohistoquímica é essencial para suspeitar do diagnóstico. Neste paciente, CK7, CD99 e BCL2 capacitaram a suspeita do diagnóstico e do tumor ser classificado como bifásico, uma vez que CK7 é encontrado em células epiteliais e CD99 juntamente com BCL2 são encontrados em células de fuso (Fig. 0.1 D-F). 4

O SS tem uma translocação patognomônica entre os cromossomos X e 18, t (X;18) (p11.2;q11.2), traduzindo-se em várias expressões diferentes de proteínas de fusão SS18:SSx; sendo SS18-SSX1 e SS18:SSX2 os mais comuns. Após a imunohistoquímica, foi possível corroborar o diagnóstico demonstrando a expressão de translocação e proteínas de fusão; a fusão é detectável em > 95% dos casos, sendo uma ferramenta-chave para confirmar o diagnóstico. 1 4

A doença metastática está presente em 24% dos pacientes no momento do diagnóstico e o prognóstico para pacientes sem metástase é favorável. No entanto, a metástase ocorre em 50 a 70% dos casos, e a maioria se desenvolve nos pulmões, seguida por osso e fígado. O tratamento padrão é a ressecção cirúrgica para SSs localizadas com a consideração do uso de radiação adjuvante e ou terapia anticâncer sistêmica. No entanto, não há uma abordagem padrão para o uso da terapia sistêmica. Quando prescritas, antraciclina mais ifosfamida são geralmente a terapia de primeira linha. No entanto, futuros ensaios clínicos definiriam um tratamento adequado para esta doença. 5 O conhecimento da doença pode ajudar a orientar a imunohistoquímica, corroborando o diagnóstico e iniciando uma terapia individualizada que leva a melhores resultados.
